# High-pressure synthesis and magnetic properties of tetragonal *R*_2_BaCuO_5_ (*R* = Sm and Eu)

**DOI:** 10.3389/fchem.2023.1166475

**Published:** 2023-06-09

**Authors:** Swarnamayee Mishra, Premakumar Yanda, Shrikant Bhat, Martin Etter, A. Sundaresan

**Affiliations:** ^1^ Chemistry and Physics of Materials Unit, Jawaharlal Nehru Centre for Advanced Scientific Research, School of Advanced Materials, Bangalore, India; ^2^ Deutsches Elektronen-Synchrotron (DESY), Hamburg, Germany

**Keywords:** high-pressure synthesis, polymorphism, antiferromagnetism, magnetocaloric effect, paramagnetism

## Abstract

We report the experimental discovery of a new structural phase of well-known orthorhombic *R*_2_BaCuO_5_ (*R* = Sm and Eu), exhibiting a tetragonal crystal structure with space group P4∕mbm . The high-pressure tetragonal phase is isostructural with the brown phase *R*_2_BaCuO_5_ (*R* = La, Pr, and Nd). In this structure, the Cu ions form an isolated square planar environment, contrary to the orthorhombic phase, where the Cu ions are located in a distorted square pyramid. Magnetization and specific heat measurements reveal the long-range antiferromagnetic order of the Cu^2+^ and/or Sm^3+^ moments for the Sm-sample, with the magnetic specific heat accounting for only 35% of the magnetic entropy. Interestingly, the Eu-sample remains paramagnetic down to the lowest temperature. The high Curie-Weiss temperature of −140 K and magnetic entropy of 3% of the expected value indicates that the system is highly frustrated. We estimated the isothermal entropy change and investigated the magnetocaloric effect for Eu_2_BaCuO_5_, and the maximum entropy change detected at a field of 70 kOe at 3 K reaches 5.6 J kg^-1^K^−1^.

## 1 Introduction

The oxides of the general formula *R*_2_Ba*M*O_5_ (*R* = rare-earth, *M* = 3*d*-transition metal) form a large family of compounds that crystallize into four different structural types with intriguing magnetic properties (Michel and Raveau, 1982; 1983 St. Schiffler and Müller-Buschaum, 1986; St. Schiffler and Müller-Buschbaum, 1986; 1987a; 1987b; Mevs and Müller-Buschbaum, 1989; Mevs and Müller-Buschbaum, 1989; Müller-Buschbaum and Sonne, 1990; Amador et al., 1990; Salinas-Sánchez, Sáez-Puche, and Alario-Franco, 1990; Salinas-Sánchez et al., 1991; Sáez-Puche et al., 1992; Salinas-Sánchez et al., 1992; García-Matres, Martínez, et al., 1993; De Andrés et al., 1993; Sáez-Puche and Hernández-Velasco, 1994; Hernández-Velasco, Salinas-Sánchez, and Sáez-Puche, 1994). Amongst these, the best known is the green phase *R*_2_BaCuO_5_ (*R* = Sm-Gd, Dy-Lu, Y) compounds exhibiting the orthorhombic structure with space group Pnma where the coordination polyhedron of copper is a distorted tetragonal pyramid CuO_5_ which is linked by monocapped trigonal prisms (*R*O_7_) by sharing triangular faces. In this structure, the Ba^2+^ ions are coordinated with eleven oxygen atoms forming an irregular BaO_11_ polyhedron. This structure is shown by most of the copper and some of the nickel, cobalt, and zinc oxides. The second kind, Nd_2_BaPtO_5_, is a tetragonal structure with space group P4∕mbm with the Pt atoms located at an isolated square planar environment PtO_4_, forming a quasi-bi-dimensional arrangement. The third kind, *R*_2_BaZnO_5_ (*R* = La and Nd), is a tetragonal I4∕mcm structure where Zn^2+^ ions are in tetrahedral coordination. The fourth one is the so-called Nd_2_BaNiO_5_ structure type showing orthorhombic symmetry with space group Immm . This structure can be described by isolated chains of corner-shared (NiO_6_) octahedra running parallel to the *a*-axis, giving rise to a one-dimensional character. In addition, there exists a dimorphism for *R*_2_Ba*M*O_5_ (*M* = Ni and Co) with smaller size *R*-ion, which crystallize in both Pnma and Immm structures depending on the synthesis conditions (Salinas-Sánchez et al., 1991; García-Matres, Rodríguez-Carvajal, et al., 1993; Hernández-Velasco and Sáez-Puche, 1993).

The *R*_2_BaCuO_5_ oxides have two different structures depending on the lanthanide trivalent cation size. For lanthanide ions Sm-Gd, Dy-Lu and Y, the structure is orthorhombic with space group *Pnma*, called the “green phase”. Whereas a tetragonal structure of cuprates, the so-called “brown phase” is observed for *R* = La, Pr, and Nd, space group *P*4/*mbm*. Table 1 shows a comparison of structural types, magnetic properties, and the ordering temperatures among *R*_2_BaCuO_5_ compounds. It can be noted that previous studies reveal some of the green phase compounds exhibited strong magnetoelectric coupling, mainly due to the interplay of 4*f*-3*d* magnetic interactions (Indra et al., 2019; Yanda, Ter-Oganessian, and Sundaresan, 2019; Yanda et al., 2020; 2021). For example, Sm_2_BaCuO_5_ demonstrates a linear magnetoelectric effect below the Cu^2+^ spins ordering temperature, and this coupling is further affected by the Sm^3+^ spins ordering (Indra et al., 2019; Yanda, Ter-Oganessian, and Sundaresan, 2019). Also, Gd_2_BaCuO_5_ is shown to exhibit multiferroic properties (Yanda et al., 2020). Further, Dy_2_BaCuO_5_ and Ho_2_BaCuO_5_ compounds evidence the linear magnetoelectric effect and field-induced multiferroicity, while Er_2_BaCuO_5_ exhibits field-induced electric polarization around 5.1 K above the critical field H_c_ ~ 0.9 T, indicating coupling between magnetism and electric polarization (Indra et al., 2019; Yanda et al., 2021). The remaining compounds in the green phase series with *R* = Eu, Y, Tm, and Lu, however, do not show magnetoelectric coupling (Yanda, 2021).

**TABLE 1:** Comparison of magnetic properties among *R*_2_BaCuO_5_ (*R* = rare-earth cations) compounds. FM and AFM mean ferromagnetic and antiferromagnetic orderings, respectively.

Compound	Structure type	Magnetic ordering type	Magnetic ordering temperature (K)		Reference
			*T*_N_^Cu^/*T*_C_^Cu^	$T_{N}^{R}$	
La_2_BaCuO_5_	P4∕mbm	FM	5.7	-	Salinas-Sánchez and Sáez-Puche, 1993
Pr_2_BaCuO_5_	P4∕mbm	AFM	20		Sáez-Puche, Herrera, and Martínez 1998; Puche et al., 2008
Nd_2_BaCuO_5_	P4∕mbm	AFM	7.8		Sáez Puche et al., 2006
Sm_2_BaCuO_5_	Pnma	AFM	23	5	Levitin et al., 1990
Eu_2_BaCuO_5_	Pnma	AFM	16	-	Levitin et al., 1990
Gd_2_BaCuO_5_	Pnma	AFM	11.9		Levitin et al., 1990
Dy_2_BaCuO_5_	Pnma	AFM	18	10	Levitin et al., 1990; Salinas-Sánchez, Sáez-Puche, and Alario-Franco 1990
Ho_2_BaCuO_5_	Pnma	AFM	17	8	Levitin et al., 1990
Er_2_BaCuO_5_	Pnma	AFM	19	5	Levitin et al., 1990
Tm_2_BaCuO_5_	Pnma	AFM	19	-	Levitin et al., 1990
Yb_2_BaCuO_5_	Pnma	AFM	16	<2	Levitin et al., 1990
Lu_2_BaCuO_5_	Pnma	AFM	19	-	Levitin et al., 1990
Y_2_BaCuO_5_	Pnma	AFM	28	-	Chattopadhyay et al., 1989

Recently, three new members of the *R*_2_BaZnO_5_ for *R* = Pr, Sm, and Eu were synthesized by a high-pressure and high-temperature method indicating polymorphism for Sm- and Eu- crystallizing in both Pnma and I4∕mcm (Ishii et al., 2020). Considering the structural phase transition from orthorhombic to tetragonal for *R*_2_BaZnO_5_ and the different cation coordination in both phases, it will be interesting to study the evolution of the crystal structure of the *R*_2_BaCuO_5_ family under pressure. Also, if it shows polymorphism, different magnetic properties can be expected for *R*_2_BaCuO_5_. Herein, we report the stabilization of the tetragonal phase of *R*_2_BaCuO_5_ (*R* = Sm and Eu) under high-pressure and high-temperature and their crystal structure and magnetic properties.

## 2 Experimental section

Polycrystalline samples of orthorhombic *R*_2_BaCuO_5_ (*R* = Sm and Eu) were prepared as starting materials by the conventional solid-state synthesis procedure starting from the stoichiometric mixture of high-purity *R*_2_O_3_, BaCO_3_, and CuO. The reactants were ground, followed by thermal treatments at 1,000°C in the air for 12 h with some intermediate grindings to homogenize the reaction products. Two different high-pressure machines were used to prepare and study the tetragonal phases. In the first step, the samples were treated with a pressure of 4.5 GPa and a temperature of 1,000°C in a laboratory-based cubic multi-anvil high-pressure apparatus. This transformed the *R*_2_BaCuO_5_ (*R* = Sm and Eu) to the tetragonal phase, which was characterized by powder x-ray diffraction at the powder diffraction and total scattering beamline P02.1 (PETRA III at DESY, Hamburg, Germany), with the wavelength of 0.20735 Å (Dippel et al., 2015). The sample detector distance (SDD) for XRD experiments was set to ∼1,000 mm. In the second step, *in situ* energy-dispersive x-ray diffraction spectra (EDXRD) at different pressure and temperature conditions were collected for orthorhombic Sm_2_BaCuO_5_. This experiment was performed in a Hall-type six-ram LVP (mavo press LPQ6 1,500–100; Max Voggenreiter GmbH, Germany) installed at the P61B beamline at DESY, Hamburg (Farla et al., 2022) using DESY standard 14/7 high-pressure assembly. Cr_2_O_3_-doped MgO octahedron was used as a pressure transmitting medium along with a tubular-resistive graphite heater. The pressure exerted at different press loads on the sample was calculated from *in situ* EDXRD data (using a MgO pressure marker) and temperature values were deduced from the Power-Temperature relationship (at the corresponding press load). The recovered sample was then characterized by a PANalytical Empyrean alpha-1 diffractometer using monochromatized Cu Kα1 radiation (λ = 1.5406 Å). The XRD pattern of all the samples was qualitatively analyzed by the Rietveld method using the program FULLPROF suite (Rodríguez-Carvajal, 1993). EDXRD profiles were analyzed using the PDIndexer software package provided by Seto Y (Seto et al., 2010; Seto, 2012). DC magnetization measurements were performed using a superconducting quantum interference device (SQUID) magnetometer (MPMS, Quantum Design). Specific heat capacity ( $C_{p}$ ) measurement was performed in the physical property measurement system (PPMS, Quantum Design) that provides temperature control and an external magnetic field.

## 3 Results and discussion

### 3.1 Crystal structure

**FIGURE 1:**
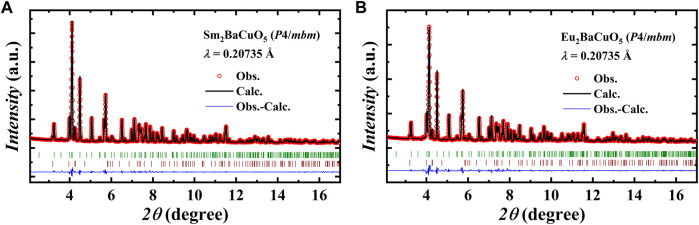
Rietveld refined synchrotron XRD pattern collected at RT of **(A).** Sm_2_BaCuO_5_ and **(B).** Eu_2_BaCuO_5_ in tetragonal phase, respectively. The second row of vertical tick marks indicates the secondary phase *R*_2_CuO_4_ (*R* = Sm and Eu).

Analysis of the room temperature XRD pattern of ambient pressure *R*_2_BaCuO_5_ (*R* = Sm and Eu) precursor, prepared by conventional solid-state synthesis, confirms an orthorhombic phase (space group Pnma ), as illustrated in Supplementary Figure S1. The corresponding refined structural parameters are given in Supplementary Tables S1, S2, which are in good agreement with the values obtained in the literature (Michel and Raveau, 1982; Salinas-Sánchez et al., 1992). The orthorhombic *R*_2_BaCuO_5_ (*R* = Sm and Eu) were subjected to high-pressure and high-temperature (HP-HT) conditions (4.5 GPa and 1,000°C). The XRD pattern of the HP-HT products obtained at the powder diffraction and total scattering beamline P02.1, with the wavelength of 0.20735 Å, is shown in Figure 1. We tried to index the data with the orthorhombic (SG: Pnma and Immm ), tetragonal (SG: P4∕mbm and I4∕mcm ) models, reported for *R*_2_Ba*M*O_5_. The tetragonal model (SG: P4∕mbm ) yielded a reliable solution consistent with the structure reported for *R*_2_BaCuO_5_ (*R* = La, Pr, and Nd).

**FIGURE 2:**
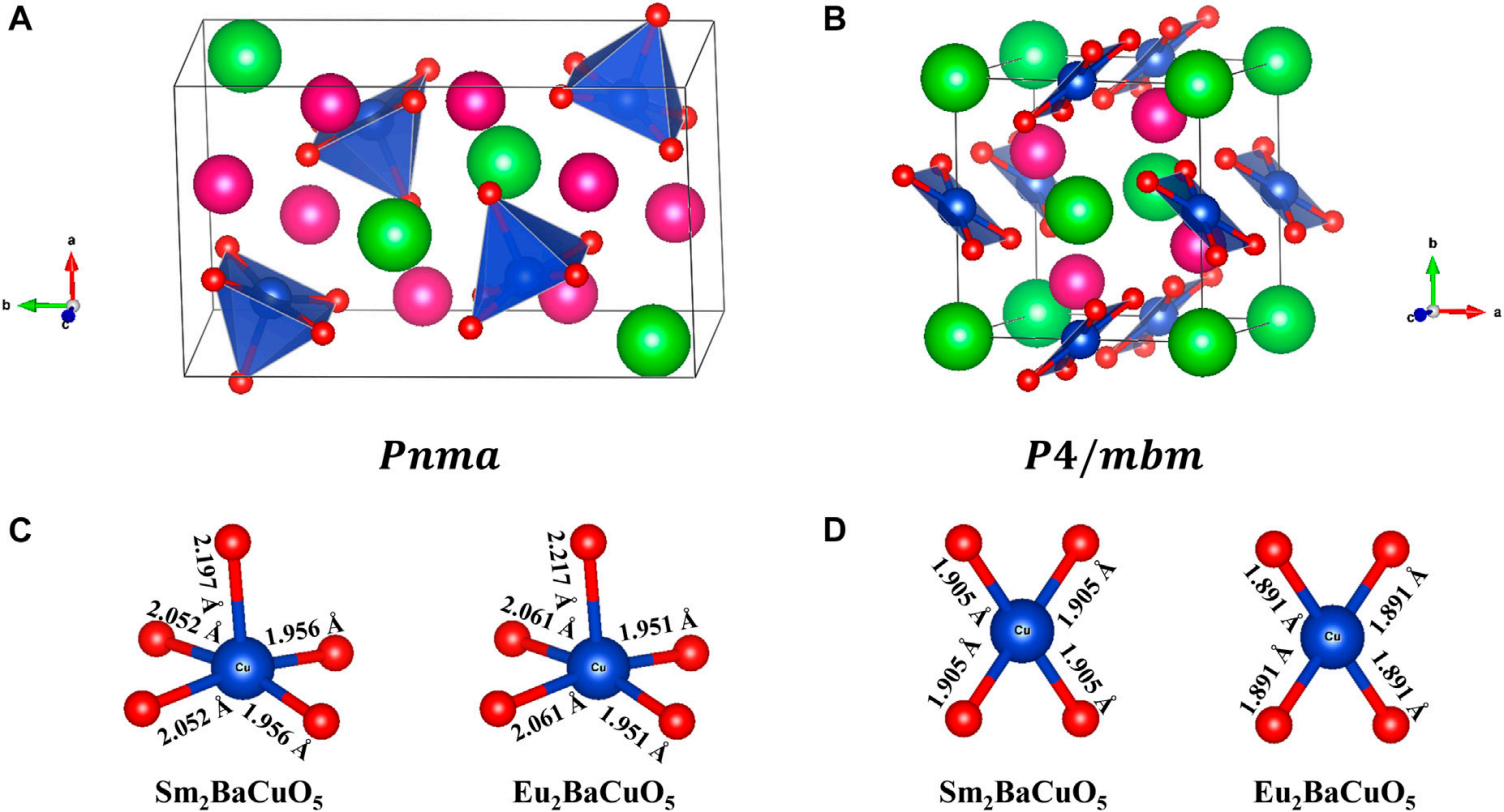
**(A, B)**. Refined crystal structure of *R*_2_BaCuO_5_ (*R* = Sm and Eu) and **(C, D)**. refined bond lengths in orthorhombic phase and tetragonal phase, respectively. (Pink: Rare-earth; Green: Barium; Blue: Copper; Red: Oxygen).

Derived atomic positions and other structural parameters of both Sm_2_BaCuO_5_ and Eu_2_BaCuO_5_ in the tetragonal P4∕mbm phase are shown in Table 2, 3, respectively. In the tetragonal phase, a small amount of Sm_2_CuO_4_ and Eu_2_CuO_4_ impurities coexist with the primary phase of Sm- and Eu- samples, respectively. In the Pnma polymorph, the rare-earth atoms are in two different crystallographic sites (Michel and Raveau, 1982; Salinas-Sánchez et al., 1992), whereas they occupy only one site in the case of P4∕mbm . As shown in Figures 2A, B, the coordination environments of the 3*d* copper atom change from CuO_5_ in the Pnma -structure to CuO_4_ square planar for the P4∕mbm -phase.

**TABLE 2:** Structural parameters obtained from Rietveld refinement of room temperature powder synchrotron XRD pattern of Sm_2_BaCuO_5_ in space group P4∕mbm . *a* = *b* = 6.6355 (3) Å, *c* = 5.7993 (3) Å, *α* = *β* = *γ* = 90 ° , V = 255.34 (2) Å^3^; and the goodness of the fit χ ^2^ = 2.45; *R*_p_ = 1.79 (%), *R*_wp_ = 2.73 (%).

Atom	Wyckoff position	Symmetry	*x*	*y*	*z*	*B*_iso_ (Å^2^)	Occ
Sm	4*h*	m . 2 m	0.1737 (2)	0.6737 (2)	0.5000	0.52 (4)	1
Ba	2*a*	4 / m . .	0.0000	0.0000	0.0000	0.50 (5)	1
Cu	2*d*	m . m m	0.0000	0.5000	0.0000	0.73 (9)	1
O1	2*b*	4 / m ..	0.0000	0.0000	0.5000	0.61 (23)	1
O2	8*k*	. . m	0.3607 (15)	0.8607 (15)	0.7611 (16)	0.35 (23)	1

**TABLE 3:** Structural parameters obtained from Rietveld analysis of room temperature powder synchrotron XRD pattern of Eu_2_BaCuO_5_ in space group P4∕mbm . *a* = *b* = 6.6052 (4) Å, *c* = 5.7900 (4) Å, *α* = *β* = *γ* = 90 ° , V = 252.61 (3) Å^3^; and the goodness of the fit χ ^2^ = 3.93; *R*_p_ = 2.38 (%), *R*_wp_ = 3.55 (%).

Atom	Wyckoff position	Symmetry	*x*	*y*	*z*	*B*_iso_ (Å^2^)	Occ
Eu	4*h*	m . 2 m	0.1736 (2)	0.6736 (2)	0.5000	0.55 (4)	1
Ba	2*a*	4 / m . .	0.0000	0.0000	0.0000	0.36 (7)	1
Cu	2*d*	m . m m	0.0000	0.5000	0.0000	0.61 (12)	1
O1	2*b*	4 / m ..	0.0000	0.0000	0.5000	0.72 (33)	1
O2	8*k*	. . m	0.3603 (22)	0.8603 (22)	0.7637 (22)	0.60 (33)	1

Further, the bond lengths for the copper polyhedra in both forms are shown in Figures 2C, D. From this, it is clear that the square pyramid CuO_5_ shows strong distortion compared to square planar CuO_4,_ which has zero distortion because of its regular shape. The distortion parameter obtained for CuO_5_ calculated with the formula

$$\nabla d=\frac{1}{n}\underset{n}{\sum }{\left[\frac{d_{n}-d_{av}}{d_{av}}\right]}^{2}$$ (1)

are 1.9 × 10^−3^ and 2.3 × 10^−3^, for Sm- and Eu- samples, respectively, where $d_{n}$ and $d_{av}$ are individual and average metal-oxygen bond lengths and n is the number of bonds in the polyhedra.

**FIGURE 3:**
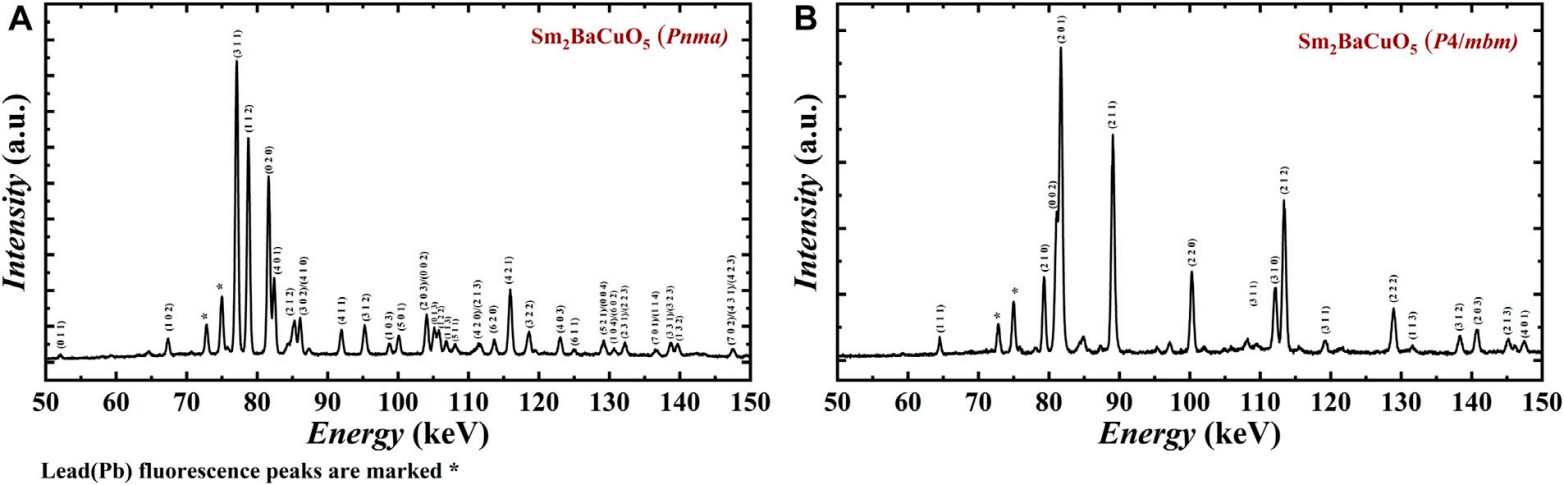
Energy-dispersive X-ray diffraction profiles of Sm_2_BaCuO_5_ analyzed at a fixed angle of 3.0230° at **(A)** ambient pressure and temperature, **(B)** recovered sample.

To investigate this high-pressure structural evolution, Sm_2_BaCuO_5_ in the orthorhombic phase was probed using energy dispersive x-ray diffraction (EDXRD), using a synchrotron white beam when the sample was exposed to different P-T conditions. The diffracted x-rays are energy analyzed at a fixed angle of 3.0230° in a continuous energy range of 30–160 keV. The obtained EDXRD spectra of Sm_2_BaCuO_5_ under ambient conditions are shown in Figure 3A, and the spectrum is consistent with the orthorhombic phase, indexed by the PDIndexer software. The sample was then subjected to pressure, and the corresponding *in situ* XRD patterns are shown in Supplementary Figure S2A. Upon compression, we observed the broadening of all reflections, and we could only detect 3–4 major broad reflections above a pressure of 1 GPa. These can be related to pressure-induced effects (amorphization or disorder state) on the sample. This effect remains consistent throughout the pressure range of 1–10.9 GPa, along with the pressure effect (peak shift). The swift change of spectra above 1 GPa also suggests the possible reorganization or disordering happens at a very early stage of the compression. On heating, reflections are getting sharper (a sign of order/recrystallization) again. The corresponding *in situ* spectra obtained at 10.9 GPa, and various temperatures are shown in Supplementary Figure S2B. It shows that the tetragonal phase appears around 720°C at 10.9 GPa. This also suggests that the orthorhombic phase transforms into disordered/amorphous state upon compression, then directly converts to a tetragonal phase upon heating. The tetragonal phase obtained at 10.9 GPa and 823°C could be retained after the quenching and recovered after decompression. The corresponding spectra are shown in Figure 3B. The sample was then crushed and measured by the laboratory angle-resolved x-ray diffraction to perform a conventional Rietveld refinement. The obtained structural parameters are enlisted in Table 4, which agrees well with the phase obtained at 4.5 GPa and 1,000°C in our high-pressure apparatus. It can be noted that at 10.9 GPa, the transition is completed within a few minutes and well below 1,000°C. We speculate that the observed phase transformation of Sm_2_BaCuO_5_ from orthorhombic to a tetragonal structure may be possible just above 1 GPa and at temperatures above 1,000°C. It is also to be noted that the tetragonal phase is stable till 10.9 GPa, supported by *in situ* experiments.

**TABLE 4:** Structural parameters obtained from Rietveld refinement of room temperature powder XRD pattern of Sm_2_BaCuO_5_ (10.9 GPa and 823 ℃ ) in space group P4∕mbm . *a* = *b* = 6.6277 (1) Å, *c* = 5.7936 (1) Å, *α* = *β* = *γ* = 90 ° , V = 254.49 (1) Å^3^; and the goodness of the fit χ ^2^ = 1.55; *R*_p_ = 3.50 (%), *R*_wp_ = 4.60 (%).

Atom	Wyckoff position	Symmetry	*x*	*y*	*z*	*B*_iso_ (Å^2^)	Occ
Sm	4*h*	m . 2 m	0.1734 (2)	0.6734 (2)	0.5000	0.36 (5)	1
Ba	2*a*	4 / m . .	0.0000	0.0000	0.0000	0.37 (8)	1
Cu	2*d*	m . m m	0.0000	0.5000	0.0000	0.32 (16)	1
O1	2*b*	4 / m ..	0.0000	0.0000	0.5000	1.000	1
O2	8*k*	. . m	0.3568 (21)	0.8607 (21)	0.7660 (27)	1.000	1

For physical property measurements, to avoid confusion for the readers, we have now named the tetragonal structured Sm_2_BaCuO_5_ and Eu_2_BaCuO_5_ synthesized at 4.5 GPa and 1,000°C in a cubic multi-anvil high-pressure apparatus installed at our lab as SBCO_HP1, EBCO_HP and the recovered Sm_2_BaCuO_5_ sample obtained at 10.9 GPa and 823°C as SBCO_HP2 in an LVP installed at the P61B beamline at DESY.

### 3.2 Magnetic properties

**FIGURE 4:**
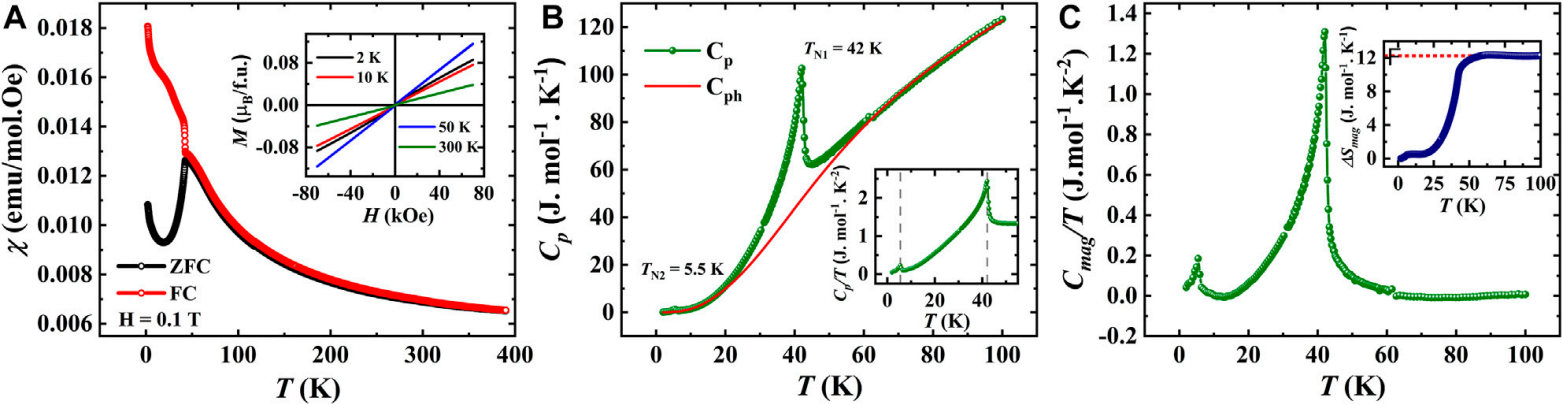
**(A).** DC magnetization as a function of temperature of SBCO_HP1 (Inset shows isothermal magnetization curves), **(B).** Temperature-dependent specific heat capacity measured in zero magnetic fields fitted with Debye-Einstein model (Inset shows *C*_p_/T vs. T), **(C).** Magnetic contribution for specific heat as a function of temperature (Inset shows a change in magnetic entropy as a function of temperature).

The temperature dependence of dc magnetic susceptibility $\chi \left(T\right)$ in an applied magnetic field of 0.1 T under both zero field-cooled (ZFC) and field-cooled (FC) procedures for SBCO_HP1 sample is presented in Figure 4A, which evidence a spontaneous change in $\chi \left(T\right)$ near 42 K and a small change near 5.5 K, indicating that the compound undergoes two successive magnetic transitions. Further information on the nature of the magnetic state is obtained from magnetic field-dependent magnetization, which was measured at four different temperatures (2, 10, 50, and 300 K), and the results are presented in the inset of Figure 4A. At 300 K, the magnetization is linear up to magnetic fields of 7 T, consistent with the paramagnetic nature. Also, the linear behavior of magnetization curves measured below 42 K, namely, at 2 and 10 K in the entire field regime, confirm the antiferromagnetic character of the compound. A splitting between ZFC and FC susceptibility branches was observed just below 42 K, which is a possible indication of the occurrence of a glassy-like magnetic behavior. Therefore, we carried out the ac magnetic susceptibility measurements on the SBCO_HP1 sample to examine the dynamic effects linked to magnetically frustrated properties and examine whether the susceptibility anomaly is associated with a glass behavior. The in-phase component M′ and the out-of-phase component M″ of the complex ac magnetization with varying frequencies are shown in Supplementary Figure S3. A sharp transition is noticed around 42 K, which agrees with the dc magnetic measurements. And this maximum has no dependence on the applied ac magnetic field frequency. It confirms that the ZFC-FC bifurcation at 42 K is not associated with the spin-glass transition and confirms the long-range magnetic ordering. However, we did not observe any features in the imaginary part.

Further, to investigate the antiferromagnetic transitions, heat capacity measurements have been carried out under zero applied magnetic field. The λ-type transitions at 42 and 5.5 K, as displayed in Figure 4B, confirm two long-range magnetic orderings. However, neutron diffraction studies are required to verify the Cu^2+^ and/or Sm^3+^ ion orderings. In order to assess the magnetic entropy changes, the phononic contribution to the specific heat has been estimated by fitting the heat capacity using the Debye-Einstein model, as shown in Figure 4B. The magnetic specific heat is obtained by subtracting *C*_ph_ from *C*_p,_ and its temperature variation is shown in Figure 4C. The magnetic entropy is further obtained by integrating the magnetic specific heat *C*_mag_/T, which yields the magnetic entropy changes 12.3 J mol^-1^K^−1^. The obtained entropy is 35% of the theoretically predicted value of $R\left[\ln{\left(S_{Sm}+1\right)}^{2}\left(S_{Cu}+1\right)\right]=$ 35.6 J mol^-1^K^−1^ for $S_{Sm}=\frac{5}{2}$ and $S_{Cu}=\frac{1}{2}$ spins, which suggests the presence of strong magnetic frustration present in the sample.

**FIGURE 5:**
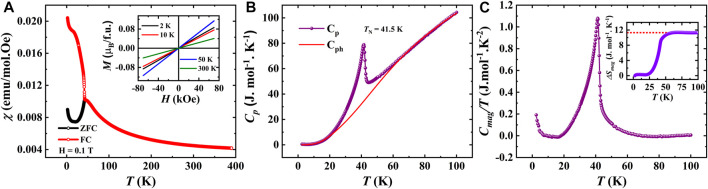
**(A)**. Temperature dependent dc susceptibility measured under 0.1 T for SBCO_HP2 (Inset shows *M*(*H*) curves measured at different temperatures), **(B)**. Temperature dependent specific heat measured under zero magnetic field and its fit with Debye-Einstein model, **(C)**. Magnetic contribution for specific heat as a function of temperature (Inset shows a change in magnetic entropy as a function of temperature).

The temperature-dependent magnetic susceptibility $\chi \left(T\right)$ in both ZFC and FC modes investigated on the SBCO_HP2 sample are shown in Figure 5A. In contrast to SBCO_HP1, these curves suggest a single AFM transition with *T*_N_ of about 41.5 K, which is supported by the heat capacity curve as displayed in Figure 5B. Subsequent isothermal magnetization curves, *M(H)*, at 2 K, 10 K, 50 K, and 300 K, are given in the inset of Figure 5A. The curves below *T*_N_ are linear and in good agreement with the antiferromagnetic ordering of Cu- and Sm-ions and room temperature data resembling the paramagnetic behavior. Further, we have fitted the heat capacity with the Debye-Einstein model, as shown in Figure 5B, to calculate the phononic contribution. In the following steps, we calculated magnetic entropy changes in the SBCO_HP2 sample as presented in Figure 5C, which gives 11.3 J mol^-1^K^−1^, almost 32% of the theoretically predicted value. It should be noted that the upturn in *C*_mag_, as shown in Figure 5C, at low temperatures suggests the suppression of the second magnetic transition under the effect of pressure.

**FIGURE 6:**
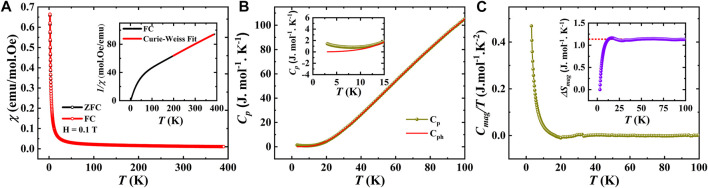
**(A)**. Temperature dependence of the magnetization measured under 0.1 T for EBCO_HP (Inset shows Curie-Weiss fit of inverse susceptibility), **(B)**. Temperature-dependent heat capacity measured at 0 T (Inset shows the zoomed view in the low-temperature region). **(C)**. Magnetic specific heat against temperature (Inset shows magnetic entropy as a function of temperature).

Figure 6A shows the temperature evolution of the dc magnetic susceptibility measured under a magnetic field of 0.1 T for EBCO_HP. The susceptibility increases with decreasing temperature without any signature of magnetic anomaly, indicating the paramagnetic behavior of this compound. This behavior is supported by the heat capacity data, which exhibits no anomalies in the temperature range of 2–100 K, as displayed in Figure 6B. Generally, for Eu^3+^-containing compounds, Eu^3+^ ions do not contribute to the magnetism and typically $\mu_{eff}\;\left(Eu^{3+}\right)=0$ . It is expected, therefore, that the ground state for Eu^3+^ may be non-magnetic. At low temperatures, $\chi \left(T\right)$ displays Van Vleck paramagnetism which is characteristic of Eu^3+^ compounds and is a result of the sole population of the non-magnetic ground state. As the temperature rises, crystal field states originating from the first excited multiplet are populated. This leads to a temperature-dependent contribution to magnetic susceptibility. Therefore, we have fitted the paramagnetic region by the Curie-Weiss law with an additional Van Vleck contribution, and the fit is presented in the inset of Figure 6A. The fit to the experimental data gives $\mu_{eff}=$ 6.23 $\mu_{B}$ and $\theta_{CW}=$ −140.1 K. The negative and high value of $\theta_{CW}$ indicates the presence of strong antiferromagnetic interaction at low temperatures. Further, as shown in the inset of Figure 6B, there was a finite heat capacity at very low temperatures. Hence, we calculated the lattice contribution from the total heat capacity using the Debye-Einstein model to extract the magnetic entropy associated with it. Figure 6C represents the temperature variation of the magnetic heat capacity, and its inset shows the change in magnetic entropy. The magnetic entropy saturates at a value of 1.1 J mol^-1^K^−1^, which is only 3% of the theoretical value for $R\left[\ln{\left(S_{Eu}+1\right)}^{2}\left(S_{Cu}+1\right)\right]=$ 38.1 J mol^-1^.K^−1^ for $S_{Eu}=3$ and $S_{Cu}=\frac{1}{2}$ spins. Hence, the compound has a small finite magnetic entropy, indicating the presence of strong short-range correlations and high magnetic frustration.

**FIGURE 7:**
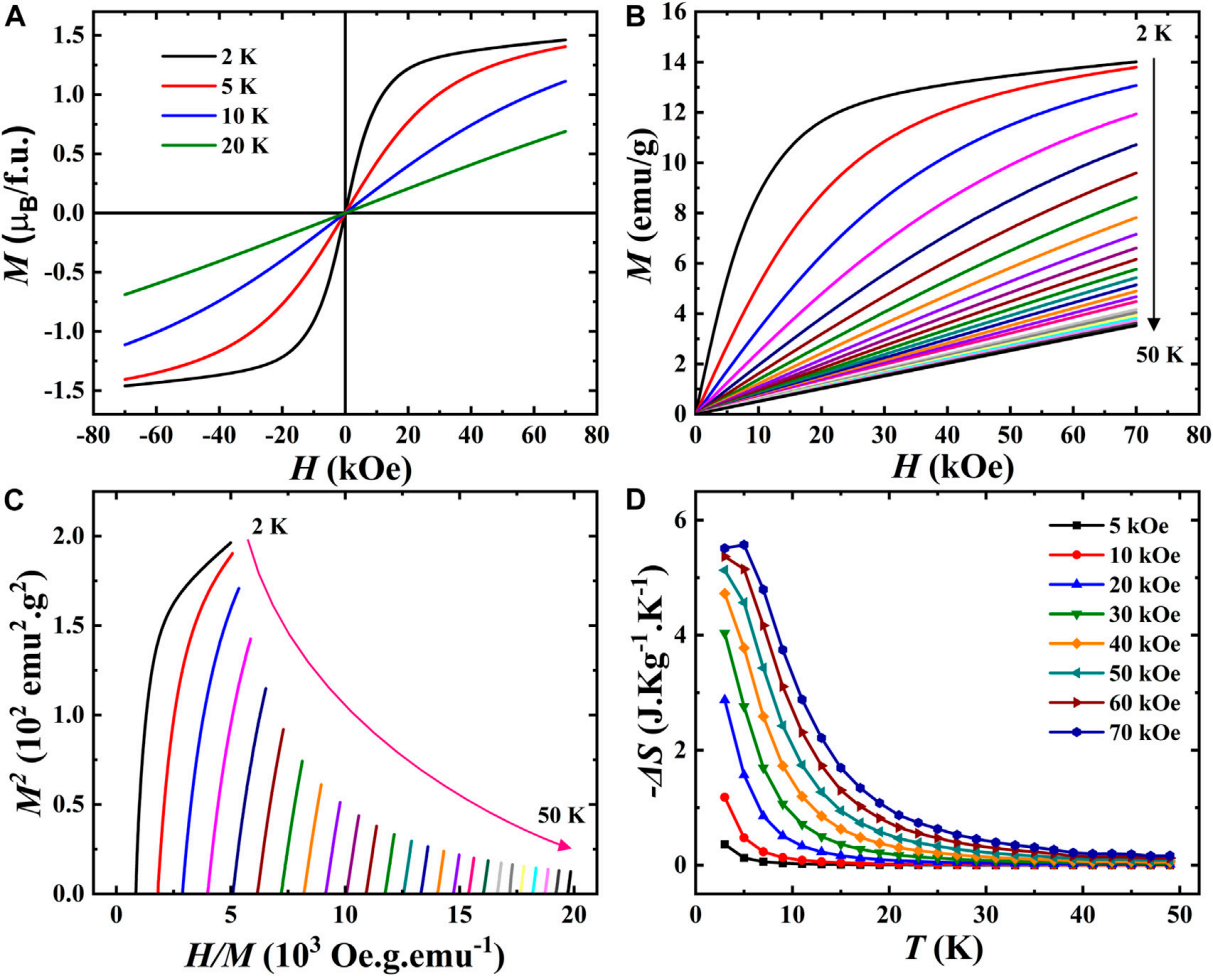
**(A)**. Isothermal magnetization against the magnetic field recorded at different temperatures for EBCO_HP. **(B)**. M(H) curves from 2–50 K measured from 0 to 7 T. **(C)**. Arrott plots. **(D)**. Temperature-dependent magnetic entropy change ( ΔS ) under different magnetic fields obtained from M vs. H data.

To investigate further the magnetic properties of this material, magnetic field dependence studies were performed. Figure 7A shows the field dependence at various temperatures for positive and negative fields up to 7 T. Interestingly, the magnetization curve recorded at 2 K shows a nonlinear behavior with a maximum magnetization of 1.5 $\mu_{B}$ /f.u at 7 T. This saturation decreases with an increase in temperature as the paramagnetic contribution becomes more prominent. Such behavior is possible because of the increased magnetic interactions between the Cu^2+^ and/or Eu^3+^ spins with temperature lowering. Considering the weak magnetic interaction between the Cu^2+^ and/or Eu^3+^ spins, we now concentrate on the temperature dependence of field-induced saturation and the associated entropy changes in the low-temperature region. Hence, we have explored the magnetocaloric effect (MCE) in this compound and calculated the respective entropy changes during isothermal magnetization processes under the influence of magnetic fields. For this purpose, we have measured single quadrant magnetic-field-dependent isotherms from 2 to 50 K with a temperature interval of 2 K, as depicted in Figure 7B. Arrott plots (M^2^ vs. H/M plots) have been used to determine the nature of the magnetic transition, which are shown in Figure 7C. According to Banerjee criterion (Banerjee, 1964), a magnetic phase transition can be first order when the slope of the Arrott curves is negative. In contrast, it will be second-order when the slope is positive. As can be seen from the figure, the slope of the Arrott plots at all the temperatures (except at 2 K) is positive, indicating that the EBCO_HP compound undergoes a second-order magnetic phase transition.

According to classical thermodynamic theory, the change in magnetic entropy ( ΔS ) produced by the variation of a magnetic field from 0 to H is calculated using the following equation:

$$\Delta S\left(T,H\right)=\int_{0}^{H}{\left(\frac{\partial M}{\partial T}\right)}_{H}dH$$ (2)

The entropy variation as a function of temperature under different magnetic fields is represented in Figure 7D as a function of temperature. The magnetic entropy decreases with increasing temperature, similar to the magnetic susceptibility variation in Figure 7B. We have found that the maximum change in entropy corresponding to a magnetic field variation of 70 kOe at 3 K is about 5.6 J kg^-1^K^−1^. Overall, these results indicate Eu_2_BaCuO_5_ in the tetragonal phase is a good candidate for magnetocaloric materials. Therefore, the different magnetic behaviors of *R*_2_BaCuO_5_ (*R* = rare-earth cations) can be explained based on the Cu-O-*R*-O-Cu interaction pathway, which apparently can control magnetic interactions.

## 4 Conclusion

We have successfully synthesized the HP-HT polymorph of the orthorhombic *R*_2_BaCuO_5_ (*R* = Sm and Eu) and studied their structural and magnetic properties. In summary, we can say that “A new form is born” under HP-HT. It is clear from the above investigations that *R*_2_BaCuO_5_ (*R* = Sm and Eu) exhibits polymorphism under pressure. These materials undergo an irreversible structural phase transition from orthorhombic (SG: Pnma ) to tetragonal (SG: P4∕mbm ) by an abrupt change in unit cell volume under pressure, in the range 0–1 GPa for Sm-sample based on the pressure-dependent EDXRD studies. The most striking difference between the orthorhombic and tetragonal *R*_2_BaCuO_5_ (*R* = Sm and Eu) structures is the Cu-O coordination environment. In the orthorhombic structure, the Cu atom is coordinated with five oxygen atoms, four of which form the base of a square pyramid. In the tetragonal structure, the Cu atom is tetrahedrally coordinated. It is the first evidence for the existence of tetragonal *R*_2_BaCuO_5_ with *R* = Sm and Eu and extends the well-known tetragonal phase for large rare-earth cations. Also, the discovered new HP-HT polymorph of the orthorhombic *R*_2_BaCuO_5_ (*R* = Sm and Eu) demonstrates the potential of high-pressure synthesis to access novel materials. Further, SBCO_HP1 and SBCO_HP2 samples exhibit antiferromagnetic interactions, while EBCO_HP shows correlated paramagnetic behavior.

## Data Availability

The original contributions presented in the study are included in the article/Supplementary Material, further inquiries can be directed to the corresponding author.
