# Supplementary material for: High-pressure synthesis and magnetic properties of tetragonal R 2BaCuO5 (R = Sm and Eu)
Source: Front Chem. 2023 Jun 9;11:1166475. doi: 10.3389/fchem.2023.1166475 (PMC10288855; doi:10.3389/fchem.2023.1166475)

Supplementary Material

High-pressure synthesis and magnetic properties of tetragonal *R*_2_BaCuO_5_ (*R* = Sm and Eu)

Swarnamayee Mishra^1^, Premakumar Yanda^1^, Shrikant Bhat^2^, Martin Etter^2^, A. Sundaresan^1^*

^1^School of Advanced Materials, and Chemistry and Physics of Materials Unit, Jawaharlal Nehru Centre for Advanced Scientific Research, Jakkur, Bangalore – 560064, India

^2^Deutsches Elektronen-Synchrotron (DESY), Notkestr. 85, 22607 Hamburg, Germany

**Correspondence:** Corresponding Author: sundaresan@jncasr.ac.in

**Supplementary Figure S1.** Rietveld refined room-temperature x-ray-diffraction pattern of **A.** Sm_2_BaCuO_5_ and **B.** Eu_2_BaCuO_5_ in orthorhombic phase.

**
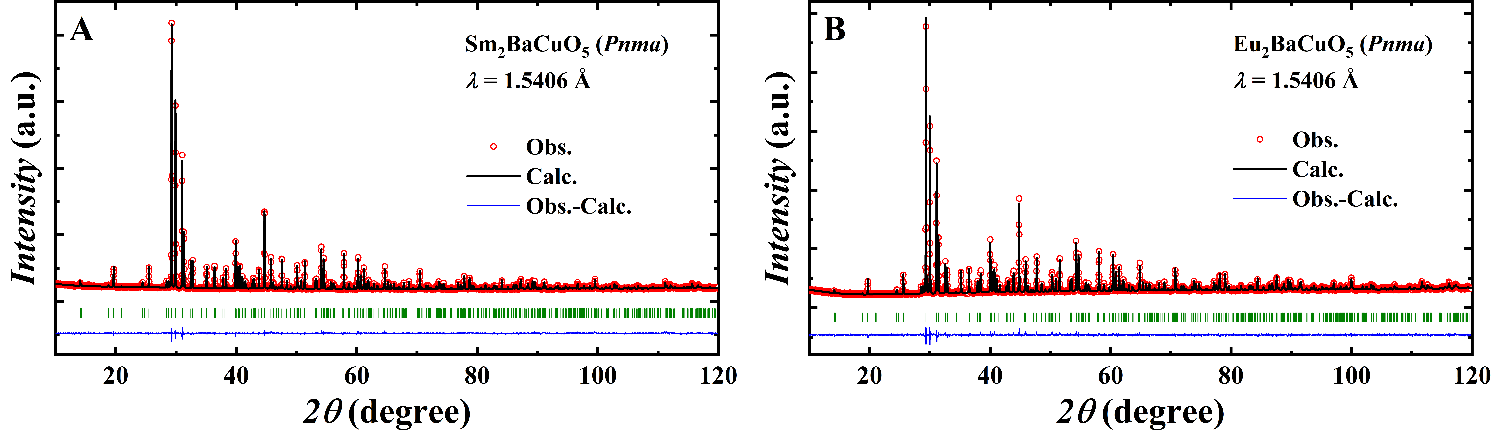
**

**Supplementary Table S1.** Structural parameters of Sm_2_BaCuO_5_ obtained from Rietveld refinement of room temperature powder XRD pattern in space group $Pnma$. *a* = 12.4046(1) Å, *b* = 5.7607(1) Å, *c* = 7.2750(1) Å, *α* = *β* = *γ* = 90$^{\circ}$, V = 519.86(1) Å^3^; and the goodness of the fit $\chi$^2^ = 1.58; *R*_p_ = 2.10 (%), *R*_wp_ = 2.66 (%).

| **Atom** | **Wyckoff position** | **Symmetry** | ***x*** | ***y*** | ***z*** | ***B*_iso_ (Å^2^)** | **Occ.** |
| --- | --- | --- | --- | --- | --- | --- | --- |
| Sm1 | 4*c* | $.m .$ | 0.2884(2) | 0.2500 | 0.1144(3) | 0.41(6) | 1 |
| Sm2 | 4*c* | $.m .$ | 0.0741(2) | 0.2500 | 0.3940(3) | 0.44(5) | 1 |
| Ba | 4*c* | $.m .$ | 0.9061(2) | 0.2500 | 0.9306(3) | 0.81(6) | 1 |
| Cu | 4*c* | $.m .$ | 0.6593(5) | 0.2500 | 0.7136(8) | 0.68(14) | 1 |
| O1 | 8*d* | $1$ | 0.4320(17) | -0.0132(28) | 0.1729(18) | 1.000 | 1 |
| O2 | 8*d* | $1$ | 0.2256(13) | 0.5079(32) | 0.3521(25) | 1.000 | 1 |
| O3 | 4*c* | $.m .$ | 0.0985(20) | 0.2500 | 0.0703(31) | 1.000 | 1 |

**Supplementary Table S2.** Structural parameters of Eu_2_BaCuO_5_ obtained from Rietveld refinement of room temperature powder XRD pattern in space group $Pnma$. *a* = 12.3673(1) Å, *b* = 5.7446(1) Å, *c* = 7.2480(1) Å, *α* = *β* = *γ* = 90$^{\circ}$, V = 514.93(1) Å^3^; and the goodness of the fit $\chi$^2^ = 1.55; *R*_p_ = 2.39 (%), *R*_wp_ = 3.10 (%).

| **Atom** | **Wyckoff position** | **Symmetry** | ***x*** | ***y*** | ***z*** | ***B*_iso_ (Å^2^)** | **Occ.** |
| --- | --- | --- | --- | --- | --- | --- | --- |
| Eu1 | 4*c* | $.m .$ | 0.2888(2) | 0.2500 | 0.1154(4) | 0.33(7) | 1 |
| Eu2 | 4*c* | $.m .$ | 0.0743(2) | 0.2500 | 0.3944(4) | 0.35(6) | 1 |
| Ba | 4*c* | $.m .$ | 0.9061(2) | 0.2500 | 0.9307(3) | 0.36(7) | 1 |
| Cu | 4*c* | $.m .$ | 0.6586(5) | 0.2500 | 0.7145(9) | 0.28(15) | 1 |
| O1 | 8*d* | $1$ | 0.4341(19) | -0.0138(33) | 0.1737(21) | 1.000 | 1 |
| O2 | 8*d* | $1$ | 0.2248(15) | 0.5068(37) | 0.3502(29) | 1.000 | 1 |
| O3 | 4*c* | $.m .$ | 0.0928(23) | 0.2500 | 0.0704(35) | 1.000 | 1 |

**Supplementary Figure S2.** Energy-dispersive x-ray diffraction profiles of Sm_2_BaCuO_5_ at **A.** different representative pressures in the range from ambient pressure to maximum 10.9 GPa at room temperature, **B.** 10.9 GPa and different temperatures.

**
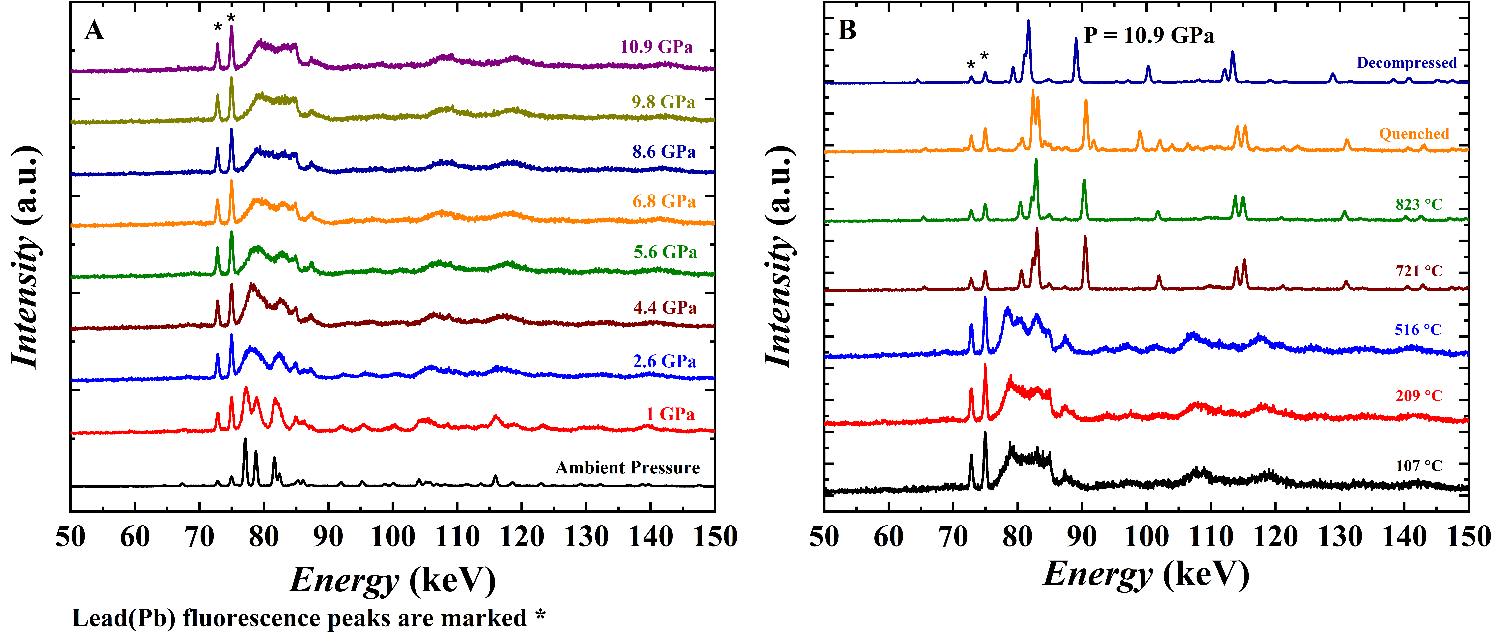
**

**Supplementary Figure S3.** Temperature-dependent **A.** real and **B.** imaginary part of ac susceptibility with different frequencies of SBCO_HP1 sample.


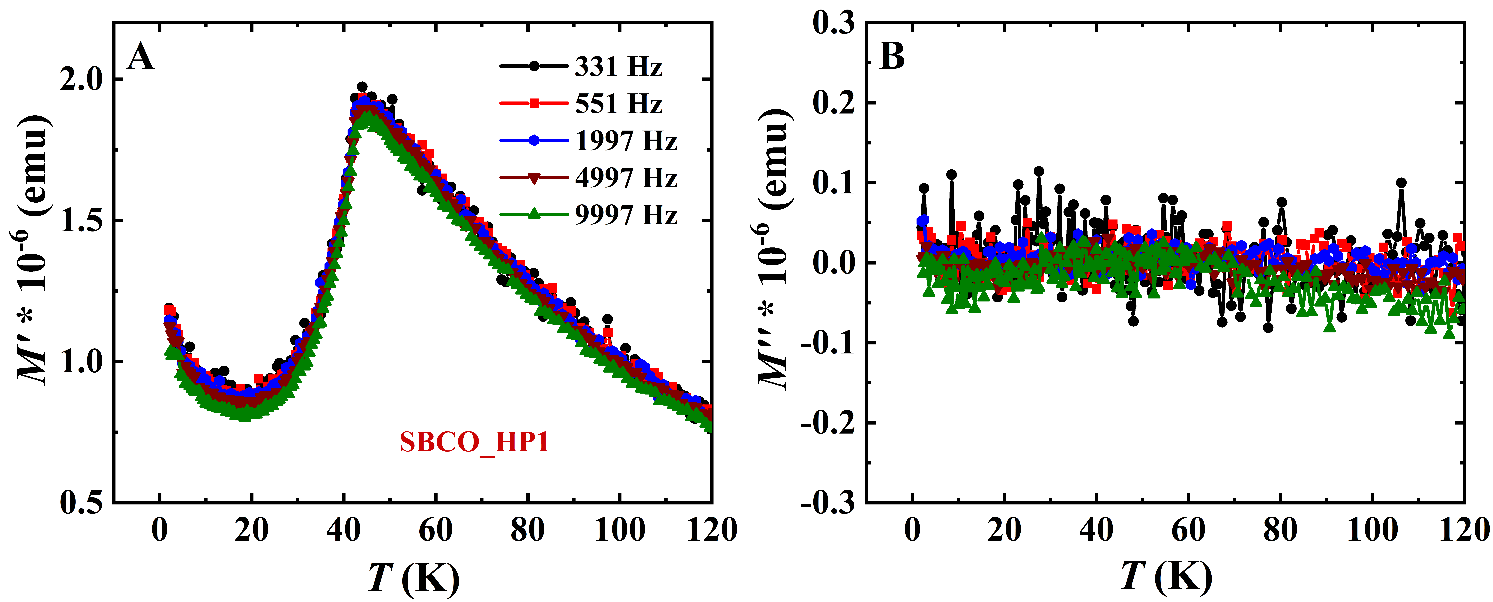

Supplement: Supplementary file 1 [file DataSheet1.docx]
